# Volatile organic metabolites identify patients with gastric carcinoma, gastric ulcer, or gastritis and control patients

**DOI:** 10.1186/s12935-017-0475-x

**Published:** 2017-11-21

**Authors:** Hongshuang Tong, Yue Wang, Yue Li, Shujuan Liu, Chunjie Chi, Desheng Liu, Lei Guo, Enyou Li, Changsong Wang

**Affiliations:** 1 Department of Anesthesiology, The First Affiliated Hospital of Harbin Medical University, No 23 Youzheng Str, Nangang District, Harbin, 150001 Heilongjiang China; 2 Department of Critical Care Medicine, Harbin Medical University Cancer Hospital, No. 150 Haping Rd., Nangang District, Harbin, 150081 China

**Keywords:** VOCs, Gastric carcinoma, Gastric ulcer, Gastritis

## Abstract

**Background:**

Gastric cancer ranks 4th among the most common cancers worldwide, and the mortality caused by gastric cancer is 2nd only to lung cancer. Gastric cancer shows a lack of specific symptoms in its early stages. In addition, its clinical symptoms often do not match the corresponding stage. Upper gastrointestinal endoscopy with biopsy is the gold standard for the diagnosis of gastric cancer because of its high accuracy. However, this operation is invasive, patient compliance is poor, and high demands for medical staff and equipment are typical of this procedure. Recent studies have demonstrated a connection between specific breath volatile organic compounds (VOCs) and various forms of cancers.

**Methods:**

We collected expired air from patients with gastric cancer, chronic atrophic gastritis or gastric ulcers as well as from healthy individuals. Solid-phase microextraction, gas chromatography–mass spectrometry and principal component analysis statistics were applied to identify potential biomarkers of gastric cancer among VOCs.

**Results:**

Fourteen differential metabolites were annotated using the NIST 11 database, with a similarity threshold of 70%. Currently, the metabolic origin of VOCs remains unclear; however, several pathways might explain the decreasing or increasing trends that were observed.

**Conclusions:**

The results of this study demonstrate the existence of specific VOC profiles associated with patients with carcinoma. In addition, these metabolites may contribute to the diagnosis and screening of patients with carcinoma.

## Background

Gastric cancer ranks 4th among the most common cancers worldwide after lung cancer, breast cancer and colorectal cancer. The mortality caused by gastric cancer ranks 2nd only to lung cancer. Every year, over 738,000 people are reported to die from gastric cancer, which accounts for 9.7% of all cancer deaths. Most cases of gastric cancer occur in developing countries, which results in an extremely heavy burden in terms of medical costs for these nations and individuals [1, 2]. Gastric cancer shows a lack of specific symptoms in its early stages. In addition, its clinical symptoms often do not match the corresponding stage. Therefore, gastric cancer is commonly characterized by a later diagnosis, poor prognosis, and likely relapse [3, 4]. A gastrointestinal barium meal examination is often an unpleasant experience for patients, and the examination itself frequently shows poor specificity. Gastric cancer involves different tissues at different stages, and serum cancer markers (CEA, CA 19-9, CA 242, CA 72-4) exhibit a lack of specificity. Upper gastrointestinal endoscopy with biopsy is the gold standard for the diagnosis of gastric cancer because it displays high accuracy. However, this operation is invasive, patient compliance is poor, and high demands for medical staff and equipment are typical of this procedure [5]. Furthermore, it is possible to overlook small lesions because the diseased areas may be patchy. In some instances, such as in the earlier stages of gastric mucosal atrophy, great inter-observer variations may be present in the identification of pre-malignant lesions. Expired air analysis, as a method for the detection of the disease course, has attracted widespread attention. *Helicobacter pylori* [14] testing is applied clinically, but a positive result can only indicate infection with *H. pylori*. Many recent studies have demonstrated a connection between specific breath volatile organic compounds (VOCs) and various forms of cancer, including lung cancer [6], liver cancer [7], breast cancer [8], and colorectal cancer [9, 10]. The reason for this connection might be that the altered blood biochemistry of cancer patients is reflected in the components of expired air through blood-air exchange in the lungs [11]. Because the expired air method demonstrates advantages such as its non-invasive nature, low cost, and good patient compliance, it has become the optimal choice for gastric cancer diagnosis [12]. Studies that address gastric cancer and related specific VOCs in expired air are scarce. Therefore, we collected expired air from patients with gastric cancer, chronic atrophic gastritis, or gastric ulcers as well as healthy individuals. Solid-phase microextraction (SPME), gas chromatography–mass spectrometry (GC–MS), and principal component analysis (PCA) statistics were applied to identify potential biomarkers of gastric cancer among VOCs.

## Methods

### Human subjects

This study included men aged between 25 and 81 years and women aged between 34 and 89 years. In addition to the group of patients with gastric diseases, this study also included healthy volunteers. As detailed in Table 1, the 24 patients with gastric carcinoma who were selected included 14 males and 10 females. The mean age of the gastric carcinoma patients was 63.75 years, with a standard deviation (SD) of 11.46 years; 8 of these patients were smokers. The 24 selected patients with gastric ulcer included 7 males and 17 females. The mean age of the gastric ulcer patients was 59.33 years, with a SD of 12.27 years; 4 of these patients were smokers. In the group of 48 patients with gastritis, 24 were male and 24 were female. Their mean age was 54.71 years, with a SD of 12.14 years; 8 of these patients were smokers. The normal control group comprised 32 individuals with a mean age of 39.78 years and a SD of 13.35 years; only one smoker was included in this group.

**Table 1:** Demographic characteristics of the study subjects

	Gastric carcinoma	Gastric ulcer	Gastritis	Normal controls
Subjects (n)	24	24	48	32
Age (mean ± SD)	63.75 (11.46)	59.33 (12.27)	54.71 (12.14)	39.78 (13.35)
Male	14	17	24	6
Female	10	7	24	26
Smokers (n)	8	4	8	1

### Breath collection

Breath gas collection with parallel collection of ambient air was performed within 24 h of overnight fasting for all the patients and control subjects.

Following gas collection, 20-ml samples of exhaled gas were drawn into gas-tight syringes (50 ml) (Agilent Inc., USA). These samples were immediately transferred to evacuated 20-ml glass vials (Supelco Inc., USA). All these vials had been thoroughly cleaned by flushing with nitrogen gas (99.999% purity, Liming Gas Inc., China) to remove any residual contaminants, after which the nitrogen gas was evacuated to allow breath sample collection. All the exhaled gas samples were analyzed within 3 h after sampling.

### Solid-phase microextraction (SPME)

A manual SPME holder with carboxen/polydimethylsiloxane (CAR/PDMS) fibers with a thickness of 75 µm was purchased from Supelco (Bellefonte, USA). The SPME fibers were inserted into the vials and exposed to the gaseous samples for 20 min at 40 °C. Subsequently, desorption of volatiles was performed in a hot GC injector at 200 °C for 2 min.

### GC/MS analysis

Analysis was performed using a GC/MS (Shimadzu GC–MS QP 2010, Shimadzu, Japan) equipped with a DB-5MS (length 30 m * ID 0.250 * film thickness 0.25 µm; Agilent Technologies, USA) plot column. Injections were conducted in splitless mode. The temperature of the injector was 200 °C. The flow rate of the helium (99.999%) carrier gas was kept constant at 2 ml min^−1^. The column temperature was held at 40 °C for 2 min to concentrate the hydrocarbons at the head of the column and was then increased by 7 °C min^−1^ to 200 °C for 1 min, followed by ramping at 20 °C min^−1^ to 230 °C for 3 min. The MS analyses were performed in full-scan mode, using a scanning range of 35–200 amu. The ion source was maintained at 230 °C, and an ionization energy of 70 eV was used for each measurement.

### Extraction and pretreatment of GC/MS raw data

Raw GC/MS data were converted into CDF format (NetCDF) files using Shimadzu GCMS Postrun Analysis software and were subsequently processed using the XCMS toolbox. The XCMS parameters consisted of the default settings with the following exceptions: xcmsSet (fwhm = 8, snthresh = 6, max = 200); retcor (method = “linear”, family = “gaussian”, plottype = “mdevden”); a bandwidth of 8 was used for the first grouping command, and 4 was used for the second grouping command. The dataset of the aligned mass ions was exported from XCMS and could be further processed using Microsoft Excel to normalize the data prior to multivariate analyses.

### Statistical analysis

Total area normalization was performed prior to statistical analysis. The normalized data were then exported to SIMCA-p 11.5 for principal component analysis (PCA) and partial least-squares discriminant analysis (PLSDA). To guard against overfitting, the default seven-round cross-validation in SIMCA-p software was applied, and permutation tests with 100 iterations were performed to further validate the supervised model. In addition, the two-sided Welch Two-Sample t-test was performed to determine the significance of each metabolite. Based on variable importance in the projection (VIP values) from the PLSDA model and P-values from t-tests with thresholds of 1 and 0.01, potential metabolic biomarkers were selected.

## Results

### Patients with gastric carcinoma versus controls

**Fig. 1:**
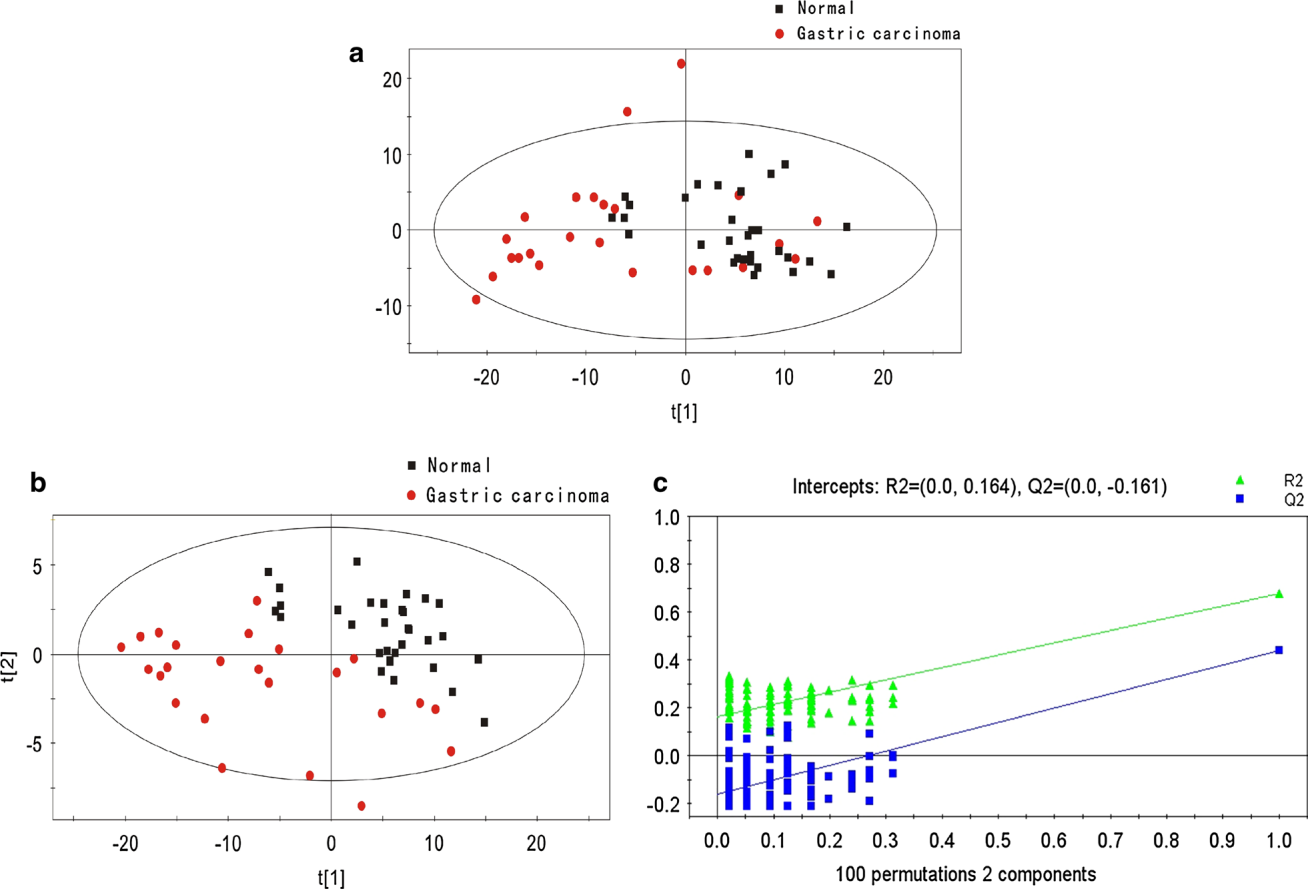
**a** PCA score plot: gastric carcinoma patients vs controls. **b** OPLSDA score plot: gastric carcinoma patients vs controls: (4 components, R2X = 0.507, R2Y = 0.676, and Q2 = 0.439). **c** PLSDA validation plot intercepts: gastric carcinoma patients vs controls R2 = (0.0, 0.164); Q2 = (0.0, − 0.161)

GC/MS was utilized to analyze the metabolites in the breath gas samples from the 24 patients with gastric carcinoma and 32 healthy controls. Based on the ion peaks in the resulting chromatogram, we obtained 215 variables. The separation trend for the experimental group and the control group was detected from the PCA and PLSDA score plots; the tight clustering of samples in the PLSDA score plot demonstrated that our approach was effective (Fig. 1).

In the corresponding PCA score plot, the exhaled air samples from the patients with gastric carcinoma and the normal controls could be separated into two different categories (Fig. 1a). To provide a more detailed explanation, PLSDA was performed. Using three orthogonal components, a prediction model was obtained (R2X = 0.507, R2Y = 0.676, and Q2 = 0.439; Fig. 1b). After 100 iterations of permutation testing, the intercept for R2 was found to be 0.164, and the intercept for Q2 was − 0.161 (Fig. 1c). In the PLSDA model, 4 characteristic metabolites played decisive roles in the sample classification, as indicated by VIP values of 0.1 and t-tests where P < 0.05 (Table 2).

**Table 2:** Related metabolites that exist at abnormal levels in the exhaled air samples among carcinoma, gastric ulcer gastritis patients and normal controls

Potential biomarker	RT	Carcinoma vs normal			Carcinoma vs gastric ulcer			Carcinoma vs gastritis		
		P-value	FC	VIP	P-value	FC	VIP	P-value	FC	VIP
2,3-Butanediol, [R-(R*,R*)]-	3.09	2.49E−03	0.8	2.1885						
1,3-Dioxolan-2-one	7.58	2.56E−03	− 1.04	2.0577						
Hexadecane	18.36	1.04E−04	3.61	1.818						
Undecane, 3,8-dimethyl-	18.36	1.30E−02	4.2	1.7379						
*N*,*N*-Dimethylacetamide	5.55				8.34E−04	− 3.45	1.9534	3.52E−11	− 3.63	2.1254
Phosphonic acid, (*p*-hydroxyphenyl)-	8.07				3.01E−04	− 1.46	1.5537	4.54E−05	− 1.12	1.6612
1,3-Dioxolane-2-methanol	11.66				4.38E−04	3.56	1.5061	2.01E−03	0.98	1.7984
3,5-Decadien-7-yne, 6-t-butyl-2,2,9,9-tetramethyl-	18.94							9.02E−08	− 11.38	2.1523
1,6-Dioxacyclododecane-7,12-dione	19.71							3.05E−07	− 5.75	1.7882
Caprolactam	15.83							2.18E−06	− 4.92	1.7731
5,7-Octadien-2-one, 3-acetyl-	14.11							7.92E−06	− 9.83	1.6588
Nonanal	11.01							2.80E−06	− 1.54	1.6415
5-Hepten-2-one, 6-methyl-	8.08							5.02E−05	− 0.96	1.6318
Benzothiazole	13.62							9.42E−03	0.64	1.5273

*RT* retention time, *FC* fold change, *VIP* variable importance in the projection

### Patients with gastric carcinoma versus patients with gastric ulcer

**Fig. 2:**
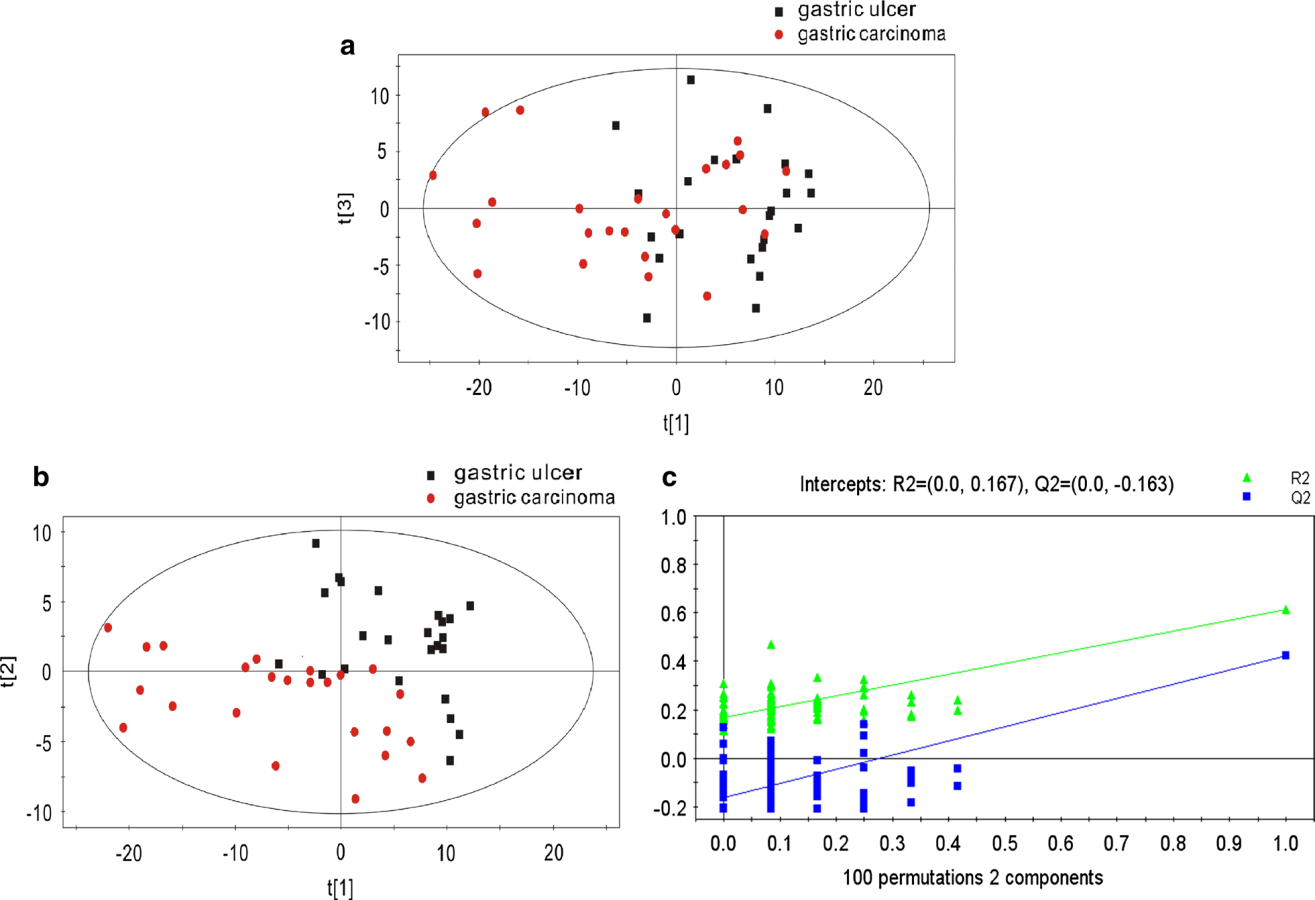
**a** PCA score plot: gastric ulcer vs gastric carcinoma patients. **b** OPLSDA score plot: gastric ulcer vs gastric carcinoma patients (3 components, R2X = 0.535, R2Y = 0.616, and Q2 = 0.423). **c** PLSDA validation plot intercepts: gastric ulcer vs gastric carcinoma patients R2 = (0.0, 0.167); Q2 = (0.0, − 0.163)

GC/MS was utilized to analyze the metabolites in the breath gas samples from the 24 patients with gastric carcinoma and 24 patients with gastric ulcer. Based on the ion peaks in the resulting chromatogram, we obtained 216 variables. The separation trend for the experimental group and the control group was detected from the PCA and PLSDA score plots; the tight clustering of samples in the PLSDA score plot demonstrated that our approach was effective (Fig. 2).

In the corresponding PCA score plot, the exhaled air samples from the patients with gastric carcinoma and the patients with gastric ulcer could be separated into two different categories (Fig. 2a). To provide a more detailed explanation, PLSDA was performed. Using three orthogonal components, a prediction model was obtained (R2X = 0.535, R2Y = 0.616, and Q2 = 0.423; Fig. 2b). After 100 iterations of permutation testing, the intercept for R2 was observed to be 0.167, and the intercept for Q2 was − 0.163 (Fig. 2c). In the PLSDA model, 3 characteristic metabolites played decisive roles in the sample classification, as indicated by VIP values of 0.1 and t-tests where P < 0.05 (Table 2).

### Patients with gastric carcinoma versus patients with gastritis

**Fig. 3:**
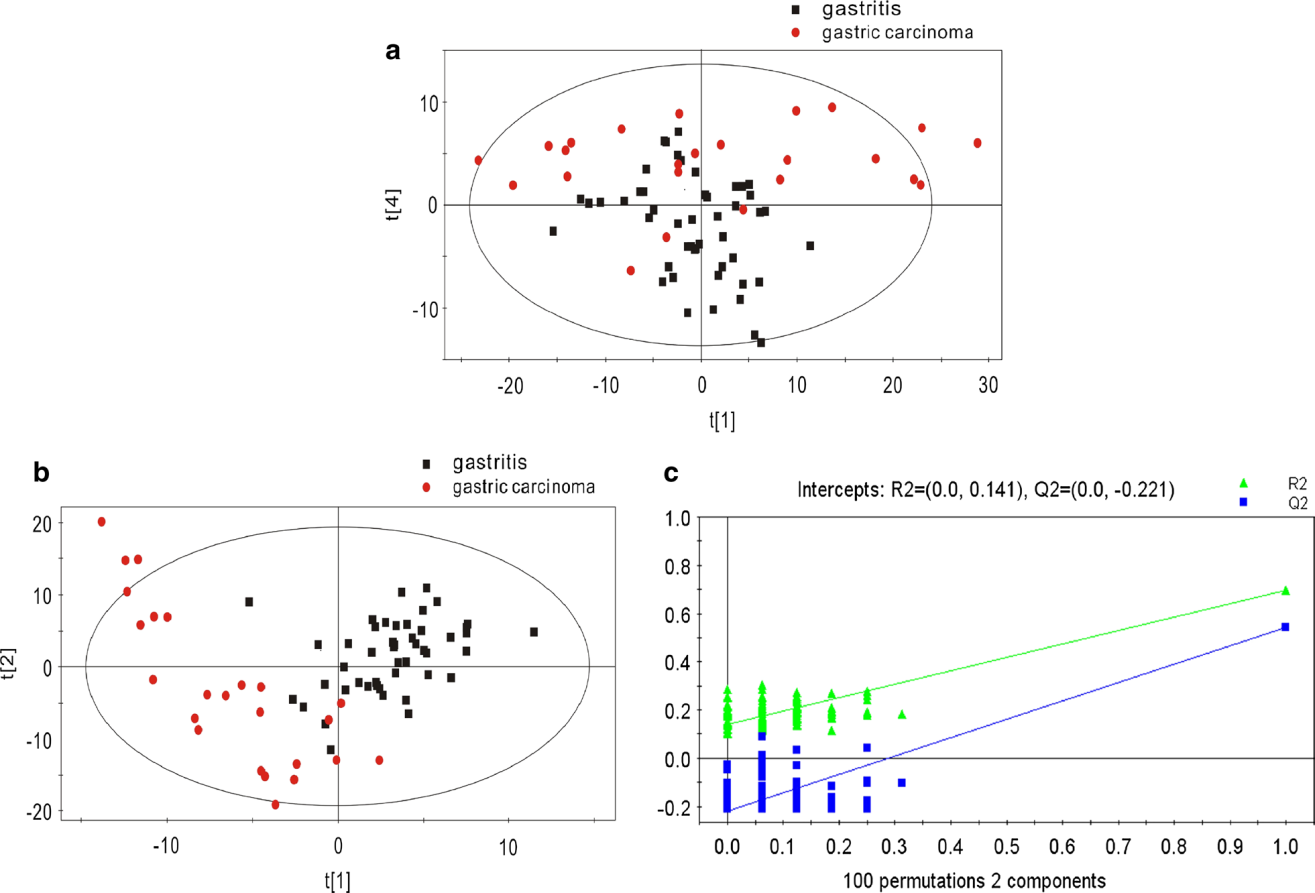
**a** PCA score plot: gastritis patients vs gastric carcinoma patients. **b** OPLSDA score plot: gastritis patients vs gastric carcinoma patients (4 components, R2X = 0.381, R2Y = 0.695, Q2 = 0.542). **c** PLSDA validation plot intercepts: gastritis patients vs gastric carcinoma patients R2 = (0.0,0.141); Q2 = (0.0, − 0.221)

GC/MS was utilized to analyze the metabolites in the breath gas samples from the 24 patients with gastric carcinoma and 48 patients with gastritis. Based on the ion peaks in the resulting chromatogram, we obtained 342 variables. The separation trend for the experimental group and the control group was detected from the PCA and PLSDA score plots; the tight clustering of samples in the PLSDA score plot demonstrated that our approach was effective (Fig. 3).

In the corresponding PCA score plot, the exhaled air samples from the patients with gastric carcinoma and the patients with gastritis could be separated into two different categories (Fig. 3a). To provide a more detailed explanation, PLSDA was performed. Using three orthogonal components, a prediction model was obtained (R2X = 0.381, R2Y = 0.695, and Q2 = 0.542; Fig. 3b). After 100 iterations of permutation testing, the intercept for R2 was shown to be 0.141, and the intercept for Q2 was − 0.221 (Fig. 3c). In the PLSDA model, three characteristic metabolites played decisive roles in the sample classification, as indicated by VIP values of 0.1 and t-tests where P < 0.05 (Table 2).

### Potential biomarkers

Among the significant metabolites identified based on the VIP values in the PLSDA model and FDR values, 14 differential metabolites were annotated using the NIST 11 database, with a similarity threshold of 70%.

The results showed that the levels of three metabolites were significantly greater in the group of carcinoma patients than in the group of normal controls (P < 0.05): 2,3-butanediol, [R-(R*,R*)]-; hexadecane; and undecane, 3,8-dimethyl-. Moreover, significantly reduced levels of 1,3-dioxolan-2-one were detected in the group of carcinoma patients compared with the normal control group (P < 0.05, Table 2).

The levels of two metabolites were significantly increased in the group of carcinoma patients compared with the group of gastric ulcer patients (P < 0.05): *N*,*N*-dimethylacetamide and phosphonic acid, (*p*-hydroxyphenyl)-. In addition, 1,3-dioxolane-2-methanol exhibited significantly reduced levels in the group of carcinoma patients compared with the group of gastric ulcer patients (P < 0.05, Table 2).

Two metabolites were found at increased levels and 8 were found at reduced levels in the group of carcinoma patients compared with the group of gastritis patients. The following metabolites were increased (P < 0.05): 1,3-dioxolane-2-methanol and benzothiazole. The following eight metabolites were found at reduced levels (P < 0.05): *N*,*N*-dimethylacetamide; phosphonic acid, (*p*-hydroxyphenyl)-; 3,5-decadien-7-yne, 6-t-butyl-2,2,9,9-tetramethyl-; 1,6-dioxacyclododecane-7, 12-dione; caprolactam; 5,7-octadien-2-one, 3-acetyl-; nonanal; and 5-hepten-2-one, 6-methyl- (Table 2).

## Discussion

*Helicobacter pylori* infection is the strongest known risk factor for the development of gastric cancer. Pylori-induced chronic inflammation may be closely related to the altered metabolic pathways of gastric cancer cells [13]. Multiple research articles have reported that inflammatory reactions are closely related to oxidative stress. The release of cytokines and the activation of immune-cell NADPH oxidase could cause an increase in reactive oxygen species (ROS) [14, 15]. Malondialdehyde (MDA) is a lipid peroxidation marker. A study by Bakan discovered that MDA levels were increased in the bodies of patients with gastric cancer [16]. Many researchers have suggested that the alkanes and methylated alkanes in the expired air of cancer patients are related to oxidative stress. One study found hydrocarbons in the breath of lung cancer patients and/or in the headspace of lung cancer cells. These hydrocarbons belonged to the following 3 families: (i) straight alkanes (pentane, heptane, octane, and decane); (ii) branched-chain alkanes (2-methylpentane, 2,3,3-trimethylpentane, 2,3,5-trimethylhexane trimethylhexane, 2,4-dimethyl-1-heptane, and 4-methyloctane); and (iii) branched-chain alkenes (2,4-dimethyl-1-heptene and 2-methyl-1,3-butadiene) [17, 18]. Another study found a significant increase in saturated hydrocarbons in the headspace of cell cultures compared with the medium of control cells. It was also shown that 2,3,3-trimethylpentane, 2,3,5-trimethylhexane, 2,4-dimethylheptane, and 4-methyloctane were released from CALU-1 cells [19]. Oxidative stress, reactive oxygen species (ROS), and free radicals are excreted from mitochondria in the cell, which generates volatile alkanes that are emitted in the breath. Many carcinogenic factors could result in increased levels of ROS in the body. ROS interact with unsaturated fatty acids in cellular and subcellular membranes, which causes lipid peroxidation and produces alkanes and methylated alkanes. The potential markers obtained in this study, including hexadecane and undecane, 3,8-dimethyl-, might be produced through this pathway. Moreover, 2,3-butanediol is one of the structural isomers of butanediol. In the human body, 2,3-butanediol is produced through the anaerobic metabolism of glucose as one of its final anaerobic metabolites [20]. In the body of gastric cancer patients, the proliferation and metabolism of tumor tissue are abnormally strong, with glucose and glutamate being used to produce energy for cancer cells and to synthesize carbohydrates, fatty acids, amino acids, and nucleotides that are needed for protein synthesis and cellular proliferation [21]. However, because the nutritional support ability of the human body is limited, normal tissues are often under chronic anaerobic conditions. In tumor tissue, glycolytic metabolism increases, which is known as the “Warburg” effect [22]. The Warburg effect can further increase glucose consumption in tumor tissue and increase anaerobic metabolism. This increase would eventually result in an abnormally high production of 2,3-butanediol as the final product of anaerobic metabolism. Similar to 2,3-butanediol, the source of alcohols in the body is the metabolism of alkanes. Cytochrome p450 enzymes in cells can catalyze the alkanes generated from lipid peroxidation to form alcohols [17]. In this study, the levels of 1,3-dioxolane-2-methanol, a potential marker, were found to be significantly increased, which might be related to the significant anaerobic metabolism of glucose, as discussed above.

In addition to the substances that showed increased levels in patients with gastric cancer, we also discovered a reduction of ketone (1,3-dioxolan-2-one 1,6-dioxacyclododecane-7,12-dione, 5-hepten-2-one,6-methyl-) and aldehyde (nonanal) levels. The decreases in these VOCs might be related to consumption due to the rapid proliferation of tumor tissues [23]. Protein metabolism in the human body can also result in ketone bodies [18]. However, in tumor tissues, protein synthesis is stronger than protein catabolism, which results in a decrease of 1,3-dioxolan-2-one 1,6-dioxacyclododecane-7,12-dione, 5-hepten-2-one,6-methyl- in expired VOCs. Using arrays of cross-reactive nanomaterial-based sensors combined with statistical pattern recognition methods, Barash et al. [19] identified and discriminated the VOC patterns of several types of lung cancer (LC) cells. These authors discovered that air from the top cell culture media in small-cell lung cancers showed decreased levels of 5-hepten-2-one, 6-methyl-, and nonanal, which was consistent with the findings of our study. Although the types of tumor cells examined in these two studies were different, the large amount of substances consumed was similar. This result explains why the concentrations of 5-hepten-2-one, 6-methyl-, and nonanal were decreased in different tumor types, including small-cell lung cancer and gastric cancer.

In a previous study, Xu et al. investigated five volatile organic compounds (2-propenenitrile, 2-butoxy-ethanol, furfural, 6-methyl-5-hepten-2-one, and isoprene) using nanomaterial-based sensors and found that those compounds were significantly elevated in patients with gastric cancer and/or gastric ulcer compared with patients with benign gastric conditions who may have similar clinical symptoms. They demonstrated that arrays of nanomaterial-based sensors can distinguish benign and malignant ulcers from other less severe gastric lesions, using breath samples of patients. They further demonstrated that the results were not affected by confounding factors such as alcohol/smoking and *Helicobacter pylori* (*H. pylori*) infection [24].

These methods are complementary for potential marker compounds identified by the SPME method and GC–MS. SPME offers some advantages as a sample preparation method of human breath, such as its high sensitivity, short extraction time, and ease of use. Because SPME extraction is based on the distribution between gaseous and liquid*/*solid phases, SPME–GC*/*MS methodology is limited by commercially available fibers to typical compound concentrations above 0.1 ppt for breath constituents [25].

In our study, by SPME method and GC–MS, 14 differential metabolites were annotated using the NIST 11 database, with a similarity threshold of 70%, among the significant metabolites identified according to the VIP values in the PLSDA model and the FDR values in patients with carcinoma compared with normal controls, patients with gastric ulcer and patients with gastritis. Our results showed that the levels of three metabolites were significantly higher in the group of carcinoma patients than in the normal control group (P < 0.05): 2,3-butanediol, [R-(R*, R*)]-, hexadecane, and undecane, 3,8-dimethyl-. In addition, significantly lower levels of 1,3-dioxolan-2-one were observed in the group of carcinoma patients compared with the normal control group (P < 0.05, Table 2). Compared with others, the more common compounds in the exhaled breath of carcinoma patients are alcohols.

Furthermore, after data processing, we obtained one potential biomarker, *N*,*N*-dimethylacetamide (DMCA), that might be a contaminant. Previous studies have suggested that this compound is present in the production process of Tedlar and is therefore a natural volatile produced from Tedlar sample collection bags [26, 27].

This study had certain limitations. (1) This was a pilot study with a small sample size. (2) No analysis was performed to distinguish between tumor stages, and many factors might affect the VOCs present in expired air such as age, sex, smoking status, and alcohol intake. Previous studies [28, 29] have shown that the components and concentrations of VOCs in various cancer stages are different. (3) No follow-up was performed among the gastric cancer patients to monitor the dynamic changes in VOCs in relation to time and the applied treatment methods. (4) The origin of volatile cancer markers in the breath may not yet correspond precisely with human physiology or pathology. In a future study, we will increase the sample size, strictly distinguish among different tumor stages, perform long-term follow-up studies to identify more accurate volatile markers, and conduct more intensive studies of the pathogenesis of volatile cancer markers in the breath.

## Conclusions

The results of this study demonstrate that specific VOC profiles are associated with carcinoma patients. In addition, these metabolites may contribute to the diagnosis and screening of patients with carcinoma.

## Abbreviations

VOCs: volatile organic compounds
SPME: solid-phase microextraction
GC–MS: gas chromatography–mass spectrometry
PCA: principal component analysis
SD: standard deviation
CAR/PDMS: carboxen/polydimethylsiloxane
PLSDA: partial least-squares discriminant analysis
ROS: reactive oxygen species
MDA: malondialdehyde
LC: lung cancer
DMCA: dimethylacetamide
